# The impact of leisure-time physical activity and occupational physical activity on sickness absence. A prospective study among people with physically demanding jobs

**DOI:** 10.5271/sjweh.4120

**Published:** 2023-11-01

**Authors:** Margo Ketels, Thomas Belligh, Dirk De Bacquer, Els Clays

**Affiliations:** 1 Department of Public Health and Primary Care, Faculty of Medicine and Health Sciences, Ghent University, Ghent, Belgium.; 2 Department of Linguistics, Faculty of Arts and Philosophy, Ghent University, Ghent, Belgium.

**Keywords:** accelerometer, physical activity health paradox, physically demanding task, sustainable employment

## Abstract

**Objectives:**

This prospective study aimed to investigate the relation between occupational physical activity (OPA), leisure-time physical activity (LTPA) and sickness absence (SA). A second aim was to explore the possible interaction effects between OPA and LTPA in determining SA.

**Methods:**

The study is based on data from 304 workers in the service and manufacturing sector. Moderate-to-vigorous physical activity (MVPA) was measured by two Axivity AX3 accelerometers for 2–4 consecutive working days. Participants reported on the level of their physically demanding tasks by using a 5-item scale from the Job Content Questionnaire. Data on SA was provided by the administration departments of the participating companies during a 1 year follow-up period. We used negative binomial regression models for our statistical analysis.

**Results:**

After adjusting for potential confounders, physically demanding tasks were significantly associated with a higher number of SA episodes and days. Accelerometer-assessed MVPA during leisure time but not during work was correlated with lower SA. Our results show a significant interaction effect between MVPA during work and leisure time in the sense that more MVPA during work increased the risk for SA days only among workers with low LTPA, but not among workers with moderate-to-high LTPA.

**Conclusions:**

Our results indicate that LTPA and OPA are related to opposite SA outcomes. MVPA during leisure time and work interact in their effect on SA, whereas we found no interaction effect between LTPA and self-reported physically demanding tasks in determining SA.

The beneficial effects of moderate-to-high levels of leisure-time physical activity (LTPA) on the reduction of cardiovascular disease (CVD) and all-cause mortality have been widely documented in the literature (1, 2). Nevertheless, growing evidence suggests that, depending on the domain of physical activity, starkly different health-related effects can be observed. Work-related or occupational physical activity (OPA) has been associated with an increased rather than decreased risk on cardiovascular diseases (CVD) and all-cause mortality (3–5). The contrasting health-related effects of these two domains of physical activity is known in the literature as the “physical activity health paradox” (3, 6).

LTPA and OPA not only seem to have opposite effects on CVD and all-cause mortality, it is also well-established that regular LTPA results in lower prevalence of sickness absence (SA) (7–9), whereas high OPA has been documented to increase the risk of long-term SA (10, 11). Physical activity and demanding postures at work have been identified as potential causes of SA, with specific actions like standing (12), forward bending of the back (13), lifting or carrying loads, and pushing or pulling loads (14) all having been linked to increased rates of SA. Capturing the relevant causal pathways and understanding the intricate network of factors that together with LTPA and OPA determine the risk of SA remains a formidable challenge. In terms of possible underlying mediating mechanisms, several possible avenues can be individuated. Environmental stressors related to a physically demanding job can lead to stress reactions, such as elevated blood pressure, which in turn is likely to cause SA (15). Jobs with higher physical demands may increase the risk of occupational injuries, and musculoskeletal disorders, eventually leading to higher SA rates. Prolonged or excessive physical exertion without sufficient rest can lead to exhaustion, also increasing the likelihood of SA.

High levels of SA is one of the significant challenges contemporary Western-European labor markets face (16). This results in substantial costs for employees, employers, organizations and society as a whole. Work-related absenteeism is estimated to cost a nation on average 2.15% of its GDP per year, amounting to €10.90 billion for Belgium in 2023 (17–18). Furthermore, there has been a steady increase in the number of SA cases over recent years (19). In 2018, 438 829 Belgian employees were on sick leave, compared to 419 940 employees in 2014 (17, 19). The prevalence of SA has been particularly pronounced among blue-collar workers, and several studies have identified high physical work demands as a main contributor to this trend (10, 12).

Given the highly adverse effects of SA on employees, employers and society at large, it is of the utmost importance to have a clear understanding of the relationship between physical activity and SA and to determine which factors might mitigate any negative impact of this relationship. Given the generally beneficial effects of LTPA on health, it is worth exploring whether LTPA might also play a role in buffering any harmful effects of OPA on SA. This has hitherto rarely been investigated, neither in the context of research on sustainable employment nor in the context of research on the physical activity health paradox. The possible interaction effects of the two domains of physical activity in determining SA remain at present poorly investigated.

With regard to measuring OPA and LTPA, previous studies have usually solely relied on self-reported measures, leading to potentially biased results in terms of validity due to social desirability, over- or underestimation due to misperception, and recall bias. It is important to keep in mind that OPA is also often defined differently across studies. Sometimes percentages of an activity are enquired about, sometimes more general questions are posed (20). It has been argued that, by using objective measures such as accelerometers, OPA can be assessed in a more nuanced way, especially for activities such as sitting, standing, walking and overall moderate-to-vigorous physical activity (MVPA) (10, 21, 22). Recent studies such as those by Gupta et al (10, 13) have paved the way for the study of the relation between SA and accelerometer-based measurements of OPA and LTPA.

Our prospective study aims to address the two aforementioned gaps and/or shortcomings in the literature and therefore investigates (i) the relation between accurately measured physical activity during both work and leisure time and SA, and (ii) whether there are any interaction effects in the relation between LTPA, OPA and SA. To do so, we rely on a combination of accelerometer-assessed physical activity and self-reported information about physical work demands. The objective measurements of physical activity are supplemented by self-reported data because specific physical demands, such as lifting heavy loads, rapid physical activity, awkward postures and lifting weights above the head, which have all been related to negative health outcomes (5, 14), cannot be captured by means of two accelerometers (22). We thus differentiate in our article between two components of OPA, namely ‘MVPA’ which comprises running, fast walking and stair walking, and ‘physically demanding tasks’, which comprise lifting heavy loads, carrying isometric loads in awkward positions, and performing awkward movements above the head or arms.

## Methods

### Study sample and design

The present study is a prospective cohort study based on data from the Flemish Employees’ Physical Activity (FEPA) study. Data were collected from February 2017 until June 2018 among 401 workers from seven different companies in Flanders. The companies were all situated in the service and production sector, ie, a logistics and courier company, a food producing company, a hospital, and four manufacturing companies. All participating workers met the following inclusion criteria: aged 18–65 years, non-pregnant, Dutch speaking, employed ≥50% of work time, and having no exclusive nightshift work. All eligible workers provided written informed consent prior to participation. The Research Ethical Committee of Ghent University Hospital approved the FEPA study (number 2017/0129), and more details about its protocol are provided in the published protocol paper (23). For the present analyses, we excluded those workers with primarily desk-based jobs, ie, sedentary jobs, resulting in a total sample of 332 physically active workers. Due to missing SA data from one company, 28 participants had to be excluded for further analysis. Thus complete SA data were available for 304 of the 332 workers. A detailed overview of the recruitment process in our study is available in the supplementary material, www.sjweh.fi/article/4120, figure S1.

### Exposure variables

*Accelerometer-assessed physical activity during work and leisure time.* Eligible participants were asked to wear two accelerometers (Axivity AX3, Axivity Ltd, Newcastle, UK), one on the right thigh and one on the back, in order to obtain measurements of their physical activity. Participants wore the accelerometers for up to 2–4 consecutive working days (24). During the measurement period, participants were asked to complete a paper-based diary reporting their working hours, time of going to and getting out of bed, periods without wearing the monitors, ie, non-wear time, and their daily reference measurement, ie, standing still in a neutral upright position for 15 seconds.

The accelerometer data were analyzed using a custom-made MATLAB program Acti4 for retrieving information with high sensitivity and specificity for various physical activities, eg, standing, walking, stair climbing and running, and certain postures, eg, sitting and standing (The National Research Centre for the Working Environment, Copenhagen, Denmark and Federal Institute for Occupational Safety and Health, Berlin, Germany) (22). Only participants with measurements for both work and leisure time for at least one valid day were included. A valid day comprised of at least 4 hours of work and 4 hours of leisure time, or at least 75% of the average reported work and leisure time. The beginning, duration and end of work and leisure time periods were derived from the information provided by the participants in their diaries.

MVPA at work was defined as the time spent running, walking on stairs, and fast walking (>100 steps per minute). The total amount of MVPA was calculated by the sum of the aforementioned activities and expressed as a percentage of the total time spent at work. The same procedure was applied to determine MVPA during leisure time.

*Self-reported physically demanding tasks.* Physically demanding tasks were assessed by using the Job Content Questionnaire (JCQ) (25). The JCQ is based on the job-demands-control-support model and is a widely used instrument to assess psychosocial and physical work demands (25, 26). Physical job demands were measured using a 5-item scale, including three specific measures of physical exertion, namely “My work requires a lot of physical effort”, “During my work, I often have to lift or move very heavy loads”, and “My work requires fast and repetitive physical exertion”, one item assessing carrying isometric loads in awkward positions, namely “I have to work frequently and for long periods in awkward or tiring body positions”, and one item assessing awkward positions above head or arms, namely “I have to work for long periods with head or arms in awkward unnatural positions”. The answers were presented on a 4-point Likert scale, ranging from 1 (totally disagree) to 4 (fully agree). A mean score over the items was calculated to obtain one overall score for physical job demands.

### Outcome variable

*Prospective register-based SA.* Officially registered SA data were gathered by the personnel administration departments of the participating companies during a 12-month follow-up period. SA data were presented as number of days (duration) and number of periods (spells) over a one-year period. SA was operationalized for our study as the accumulated number of days and periods on sick leave during the one year follow-up period.

### Covariates

Detailed socio-economic and health-related information was obtained through a questionnaire and medical screening. Age (continuous variable: years), sex (binary: male/female), educational level (1=primary school, 2=secondary school, and 3=high school or university), currently smoking (binary: yes/no) and measured body mass index (BMI, continuous variable: kg/m^2^) were included as covariates in our analysis. The JCQ was used to measure psychological job demands (5 items) and job control (9 items) including skill discretion and decision authority. Participants were categorized into two groups based on the median split of both JCQ scales: those who perceived high job strain (high demands combined with low control), and those who fall under the categories of low and no job strain.

### Statistical analysis

Before conducting further analyses, the distribution of all parameters was checked and boxplots were used to detect possible outliers. The normality of the distributions of all continuous variables was examined using the Shapiro-Wilk test. The distribution of continuous variables which are not normally distributed is presented by means of medians and interquartile ranges (IQR). Other, normally distributed, characteristics are expressed by frequencies (%) and means (standard deviations, SD). Since the outcome variable under investigation is a count variable, ie, how often SA days and periods occurred, both standard Poisson regression and negative binomial regression could be used. Standard Poisson models have somewhat restrictive assumptions regarding the distribution that can be rather easily violated, such as extra-Poisson variation. When the observed variance exceeds the predicted mean, this results in overdispersion, violating one of the assumptions of standard Poisson models. In our case, we found SA days to be overdispersed (variance/mean=59.97). We subsequently assessed whether there was an excess of zeros, ie, the occurrence of more zeros than expected, on the basis of the distribution used for modelling. Our analysis showed that the ratio of excess zeros was within the tolerance range (1.03), suggesting that a negative binomial regression model could be used as an alternative. To compare the fit of different regression models, we calculated the Akaike Information Criterion (AIC) for the standard Poisson model, the negative binomial model, and the zero-inflated negative binomial model for both SA days and SA periods. The AIC values indicated that the negative binomial model provided the best fit, with AIC values of 1703.85 and 888.06 for SA days and SA periods, respectively. By contrast, the AIC values for the zero-inflated negative binomial model were 1707.85 and 891.66, and for the Poisson model were 8867.74 and 914.64. Therefore, we used a negative binomial regression model to estimate the incidence rate ratios (IRR) with 95% confidence intervals (CI), which represent the expected increase or decrease in the number of SA days/periods.

We built the statistical models in three steps. Model 0 was the crude model. Model 1 was adjusted for age and sex. Model 2 was additionally adjusted for BMI, education, smoking, and job strain. To examine the possible interaction effects between OPA and LTPA in determining SA, an interaction term was created. For each model, the coefficient (β, ie, effect size), incidence risk ratios (IRR) with 95% confidence intervals (CI) were determined. The IRR was calculated by taking the exponential function of the coefficient estimate (e^β). IRR >1 represent an increased risk while risk ratios <1 represent a decreased risk. In our study, we employed terciles as a method to visualize the interaction effect. Terciles allow to divide data of MVPA leisure time into three equal groups based on a specific variable of interest. The significance level was set at P<0.05. All statistical analyses were conducted in R (version 3.6.2).

### Sensitivity analysis

In order to account for the fact that differences in level of education (an important socioeconomic status-related parameter) and differences in job sector of the participants might play a role in determining the (strength of the) relation between OPA, LTPA and SA, we conducted two sensitivity analyses. To do so, model 2 was executed with one stratification analysis for level of education (two levels: low-to-medium education and high education) and one stratification analysis for job sector (two sectors: service and manufacturing sector). The categorization of the job sector of the participants was based on revision 8 of the International Standard Classification of Occupations (ISCO-08) system (27). The service sector includes technicians and associate professionals, as well as service workers and sales workers. The manufacturing sector includes (i) skilled workers, eg, craft and related trades workers such as plumbing, welding, electrical work, masonry, (ii) factory workers, eg, plant and machine operators and assemblers, and (iii) unskilled workers, eg, cleaners, helpers, doorkeepers, porters, building caretakers, and hand packers.

## Results

### Descriptive characteristics

Table 1 shows that 57.2% of the participants in our sample were women. The mean age was 39, range 20–65, years. Half of the sample consisted of higher educated participants. During the first year of follow-up, 61.2% of the workers experienced ≥1 day of SA. The median of SA days during that period was 2 and the median of SA periods was 1.

**Table 1:** Descriptive characteristics (N=304). [SD=standard deviation; IQR=interquartile range; Min=minimum; Max=maximum; MVPA=moderate-to-vigorous physical activity; SA=sickness absence.]

Basic characteristics			Mean (SD)	Median (IQR)	Min–Max	N (%)
Age (years)			39 (11.3)		20–65	
Sex						
	Female					176 (57.9)
	Male					128 (42.1)
Educational level ^a^						
	Low					50 (16.4)
	Medium					98 (32.2)
	High					156 (51.3)
	Body mass index (kg/m^2^)		25 (4.2)		18–53	
Job type (sector)						
	Service sector					161 (53.1)
		Technicians & associate professionals				156 (51.3)
		Service & sales workers				5 (1.6)
	Manufacturing sector					142 (46.9)
		Skilled worker				23 (7.6)
		Factory worker				102 (33.6)
		Unskilled worker				17 (5.6)
Work schedule						
	Shift					199 (65.9)
	Day job					103 (34.1)
Work hours per week			36.8 (6.3)		8–64	
Psychological job demands ^b^			2.5 (0.5)		1.4–3.8	
Job control ^b^			2.8 (0.5)		1.22–4	
Job strain (categorical variable)						91 (30.1)
Physically demanding tasks ^b^			2.4 (0.6)		1–4	
Accelerometer-assessed information						
	Valid accelerometer wear-days		3.0 (0.9)		1–5	
	Total work time (min/day)		474 (73.2)		240–720	
	Total leisure time (min/day)		466 (100)		75–1104	
	Percentage MVPA at work		14.4 (7.3)		0–39.60	
	Percentage MVPA at leisure time		9.7 (5.0)		0–29.26	
SA (during 1 year)						
	Number of SA days			2 (0–8)	0–196	
	Number of SA periods			1 (0–2)	0–10	

^a^ Low=primary school; medium= secondary school and/or 1–2 years of specialization; high= university or university college . ^b^ 1-4 Likert scale

### Correlations between exposure variables

The results of the Pearson correlation between exposure variables showed a weak significant association between MVPA and physical demands at work (r=0.22; P<0.001), no significant association between MVPA at work and during leisure time (r=0.06; P=0.30) and also no significant association between physical demands and MVPA during leisure time (r=0.05; P=0.42).

### Negative binomial regression model

Table 2 shows the outcomes of the negative binomial regressions used to analyze the associations between OPA, LTPA and SA days. MVPA during work was not statistically correlated to SA days, whereas the self-reported physically demanding tasks did show a positive association with SA days. The fully adjusted model indicated that if workers would increase their physically demanding tasks with 1 unit on the Likert-scale, their amount of SA days would be expected to increase by a factor of 1.65, while holding all other variables in the model constant. By contrast, MVPA during leisure time showed a negative association with SA days. The fully adjusted model indicated that a one unit increase in MVPA during leisure time would be expected to decrease the amount of SA days by a factor of 0.94.

**Table 2:** Negative binomial regression analyses: the association between occupational physical activity (OPA)/leisure time physical activity (LTPA) and the number of sickness absence days, adjusted for covariates. [MVPA=moderate-to-vigorous physical activity; IRR=incidence risk ratios; CI=confidence intervals]. **Significant associations at P<0.05 are in bold.**

	Unadjusted model			Model 1 ^a^			Model 2 ^b^	
	Coeff	IRR (95% CI)		Coeff	IRR (95% CI)		Coeff	IRR (95% CI)
MVPA work	0.02	1.02 (0.99–1.05)		0.02	1.02 (0.99–1.06)		0.01	1.01 (0.98–1.04)
Physical demands	0.43	1.55 (0.99–2.42)		0.43	1.54 (0.98–2.44)		**0.51**	**1.67 (1.10–2.53)**
MVPA leisure	**-0.05**	**0.95 (0.91–0.99)**		**-0.09**	**0.92 (0.87–0.97)**		**-0.06**	**0.94 (0.89–0.99)**

^a^ Adjusted for age and sex. ^b^ Adjusted for age, sex, body mass index, smoking, educational level, and job strain.

Table 3 shows the outcomes of the negative binomial regressions used to analyze the associations between OPA, LTPA and SA periods. Table 3 shows that MVPA during work was not correlated with the number of SA periods. Physically demanding tasks during work were positively correlated with the number of SA periods. The fully adjusted model shows that an increase with 1 unit of physically demanding tasks results in an expected increase of the number of SA periods by a factor of 1.28. By contrast, MVPA during leisure time showed a trend towards negative association with SA periods. The fully adjusted model indicated that a one unit increase in MVPA during leisure time is expected to decrease the number of SA periods by a factor of 0.96 (Model 1).

**Table 3:** Negative binomial regression analyses: the association between occupational physical activity (OPA)/leisure time physical activity (LTPA) and the number of sickness absence periods, adjusted for covariates. [MVPA=moderate-to-vigorous physical activity; IRR=incidence risk ratios; CI=confidence intervals]. **Significant associations at P<0.05 are in bold.**

	Unadjusted model			Model 1 ^a^			Model 2 ^b^	
	Coeff	IRR (95% CI)		Coeff	IRR (95% CI)		Coeff	IRR (95% CI)
MVPA work	0.01	1.01 (0.99–1.03)		0.001	1.01 (0.99–1.03)		0.01	1.01 (0.99–1.02)
Physical demands	**0.24**	**1.27 (1.03–1.56)**		**0.28**	**1.32 (1.06–1.64)**		**0.25**	**1.31 (1.06–1.62)**
MVPA leisure	**-0.04**	**0.96 (0.94–0.99)**		**-0.04**	**0.96 (0.93–0.99)**		-0.02	0.98 (0.95–1.00)

^a^ Adjusted for age and sex. ^b^ Adjusted for age, sex, body mass index, smoking, educational level, and job strain.

**Figure 1:**
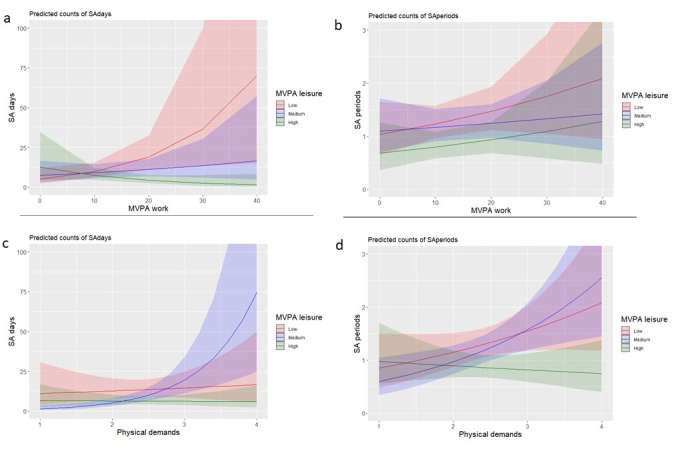
Visual representation of the interaction models of the association between OPA and MVPA during leisure on SA days and SA periods (4 separate models).

After adjustment for all confounders, a significant interaction effect between occupational MVPA, MVPA during leisure time and SA days was found (P<0.001). In order to better understand this significant interaction effect, MVPA during leisure time was divided into three equal groups (ie, terciles), as displayed in figure 1. Figure 1a, ie, which relates to the significant interaction effect, shows that occupational MVPA increases the risk for SA days the most for those workers with low MVPA during leisure time, and far less so for highly active workers in their free time. No other interaction effects turned out to be statistically significant.

### Sensitivity analyses

The results of the sensitivity analyses can be found in supplementary tables S1–4. For 5 out of 6 of the possible effects of physically demanding tasks, MVPA leisure time, and MVPA during work on SA days and SA periods, no interaction effects related to education level or job sector were found. Stratifying for level of education led to the finding that the positive association between MVPA leisure time and SA periods turned out to be only significant for the high level of education group and not for the group with low-to-medium levels of education. Stratifying for job sector led to the finding that the positive association between MVPA leisure time and SA periods (significant interaction effect) turned out to be only significant for the service and not the manufacturing sector.

## Discussion

*Main effects: the physical activity health paradox.* Our results are generally in line with the pattern associated with the physical activity health paradox. First of all, we found a negative association between the amount of MVPA during leisure time and SA, meaning that increasing MVPA during leisure time may contribute to lowering the risk of experiencing SA days and periods. Our results are in line with the results of previous studies using either self-reported measures (8, 28) or objective accelerometer data (10). With regard to the possible mediating steps underlying the beneficial effects of LTPA on reduced SA, it can be pointed out that LTPA probably improves overall health and physical capacity, thereby enabling workers to perform their work tasks better (29). Encouraging and enabling workers to move more during leisure time can therefore be recommended. Given that even a slight decrease in SA rates can result in substantial economic savings for businesses and society alike, increasing MVPA during leisure time should be fully supported and enabled by employers and societal actors.

We found no significant correlations between MVPA during work and SA, while we did find a significant positive association between physically demanding tasks and SA. This seems to indicate that physically demanding tasks in particular may contribute to more SA days and periods. The fact that objectively measured MVPA at work was not associated in our data with more SA days and periods is not in line with the findings from Gupta et al (10). They reported that spending more time on MVPA during work relative to other work-related behaviors was positively associated with long-term SA. The fact that our results indicate that performing physically demanding tasks increases the likelihood of having more SA absence days and periods is fully in line with evidence reported by Gupta et al (10), Andersen et al (30), and Andersen et al (31). These studies concur that an increase in physically demanding tasks is a relevant risk factor for SA levels.

We can proffer three hypotheses to account for the association found for physically demanding tasks but not occupational MVPA. First, as outlined in the introduction, accelerometers allow to capture global physical activities, such as walking, running, and stair climbing, but not specific activities such as awkward postures and heavy lifting. Activities such as these have been shown to have the highest correlations with negative health outcomes (12, 30, 31), potentially explaining the higher SA numbers. Second, it could be that MVPA during work only accounts for a small proportion of the work-related physical behaviors and hence has an impact too small to be detected. Third, it needs to be pointed out that, due to the self-reported nature of the information on physically demanding tasks, the difference in results could potentially be partially explained in terms of a self-report bias. Participants with lower resources might over-report their physically demanding tasks, due to a fatigue-distorted perception of these demands. This might lead to an overestimation of the strength of the association between physically demanding tasks and SA.

### Sensitivity analyses

In our analyses we also aimed to adjust for socioeconomic status, taking into account its possible role in the complex interplay of factors determining the impact of OPA and LTPA on SA. Our stratification analyses for the level of education, which can be seen as a highly useful and reliable socioeconomic indicator (32), did not point in the direction of a different relation between OPA and SA according to educational level. Also with regard to the relation between LTPA and SA days, we did not find a different relation. The only difference we found relates to the relation between LTPA and SA periods, where the relation was only significant for the high education group and not for the group with a low-to-medium level of education.

### Interaction effects

Our results showed a significant interaction effect between MVPA both during work and leisure time on SA days (P<0.001). This points to the fact that participants who are inactive during leisure time and who experience high MVPA during work face the highest risk of SA days. This finding is largely consistent with the ones reported in a recent systematic review (33). The review showed that engaging in LTPA was consistently protective among all workers, independent of their level of OPA, with regard to cardiovascular mortality and metabolic syndrome. On the other hand, for outcomes such as all-cause mortality, CVD, musculoskeletal pain, diabetes, and depression, the same systematic review indicated that the protectivity of LTPA decreased for those who experience moderate-to-high levels of OPA.

In our data, we found no evidence for an interaction effect in the relation between MVPA during leisure time and physically demanding tasks in determining the risk of SA days and SA periods (both P>0.20). Both SA days and periods can be expected to increase in function of an increase in physically demanding tasks and a decrease for MVPA during leisure time, independent of the level of each other. Our results are in line with the ones reported by Clays et al (4) and Holtermann et al (34). Both studies also failed to find an interaction effect between LTPA and physically demanding tasks in determining cardiovascular (34) and all-cause mortality (4, 34).

In general, the limited number of studies investigating the interaction between OPA and LTPA so far have produced mixed results (4, 34, 35). Most studies have found a protective effect of moderate-to-high LTPA for CVD and mortality among workers exposed to high OPA (34). On the other hand, the study of Clays et al (35) found that the combination of high OPA (in this study referring to physically demanding tasks) and high LTPA can result in an increased risk of CVD. This suggests that engaging in heavy physical activity both during work and leisure time may impose an excessive strain on the cardiovascular system, potentially accelerating the process of arteriosclerosis and thereby increasing the risk on CVD.

### Implications

Whether individuals with physically demanding jobs should prioritize rest or engage in physical activity during their leisure time remains a subject of scientific debate. Comparing results across various studies on this topic has proven to be challenging due to significant differences in methodological approaches, including variations in sample composition (our study focused solely on individuals with physically demanding jobs, whereas other studies tackled both sedentary jobs and blue-collar workers), disparate methods for assessing OPA and LTPA (self-reported versus objectively measured physical activity versus mixed methods), and differences in outcome measures (cardiovascular mortality, all-cause mortality, musculoskeletal problems, sickness absence, etc.). Our data support the line of thinking that speaks in favor of promoting LTPA, regardless of the level of OPA. In fact, our data indicate that LTPA and OPA are associated with opposite outcomes for SA and that a lack of LTPA can be detrimental to those workers experiencing high levels of OPA.

Increasing LTPA in the life of workers with physically demanding jobs is not only beneficial for the health of the workers involved, but also for the companies they work in and for the overall sustainability of the welfare state. Providing opportunities at work to practice sports, creating work schedules that allow for enough leisure time to engage in LTPA, and informing employees about the health benefits of LTPA are among the organizational factors that can enable workers to engage in physical activity in a healthy way.

### Strengths and limitations of the study

Our study has a number of strengths. First, the use of objective accelerometer measures to assess physical activity is a major improvement over various previous studies, as it allows to accurately differentiate between several types of physical behaviors during work and to avoid self-reported bias. In order to address the potential shortcoming that accelerometer measures may miss important aspects of some specific physical behaviors, such as lifting a heavy object, we complemented the use of accelerometers with self-reported questionnaires to assess the nature of some of the physically demanding tasks. Second, our sample of 304 workers is relatively large, had a more or less balanced ratio between men and women and covered the entire working age range. Third, this study is, to our knowledge, the first to investigate the interaction effects of OPA and LTPA on SA. Fourth, we took into account multiple confounders to ensure the validity of our models. Fifth, by relying on the reports from the companies, we had objective recordings at our disposal that have more coverage, accuracy and consistency compared to self-reports. Sixth, with regard to the statistical analysis of our SA data, we dealt with the potential issues of excessive zeros, overdispersion and right skewness of the data by implementing the negative binomial model.

A number of limitations need to be taken into account as well. First, since we used an observational prospective cohort design, we can only start exploring the causal dynamics at play without being able to confirm them. A second limitation is that the participating companies and participants were recruited by means of convenience sampling, which might lead to a potential selection bias at the company level. A third limitation is that a healthy worker effect may have distorted the findings. In fact, workers who are able to work and continue working in a challenging work environment are usually the ones that have the necessary physical and mental resilience to carry on in that particular context. This potential bias would lead to an underestimation of the strength of the association, as the respondents would be healthier, and possibly have experienced less SA than the non-respondents. Fourth, although multiple companies from the industry and healthcare sector were included in this study, we cannot claim that these companies constitute a perfectly representative sample of the overall economic sector. Fifth, our data that involve time spent on certain activities are compositional in nature, meaning that spending more time on one activity inevitably leads to less time spent on another activity. Compositional data analysis (CoDA) allows to capture this aspect of the data in a more correct way but was not implemented in our analyses. Sixth, although it is often presented as an acceptable threshold to reliably assess physical activity levels, conducting measurements with accelerometers for three consecutive working days can be considered a further limitation of the adopted research methods (36). This somewhat short measurement period might not accurately capture the variability in physical activity levels across different days of the week or might not account for potential changes in activity patterns due to factors such as weather, the work demands, or personal schedules. Additionally, the data gathered on a rather limited measurement window might not adequately represent an individual’s typical physical activity behavior, potentially leading to an incomplete or skewed understanding of overall activity levels, most likely leading to attenuation of the true associations.

### Concluding remarks

Our study suggests that engaging in MVPA during leisure time can lower the risk of SA days and periods. By contrast, we did not find a significant association between MVPA during work and SA. Exposure to physically demanding tasks at work was associated with increased SA days and periods, underscoring the role of physical workloads as a risk factor for workers with physically demanding jobs. We furthermore found that high MVPA during work and being inactive during leisure time is associated with the highest risk of SA days. Taken together, these results they suggest both a direct and moderating role for physical activity during leisure time. However, more prospective studies are needed to confirm the potential benefits of leisure time physical activity as a means to reduce SA and to identify effective strategies for lowering the negative outcomes associated with physically demanding tasks. Future research would do well to incorporate CoDA analyses in the investigation of the impact of different types of physical activities during work or leisure time in a 24-hour perspective on health-related outcomes.

### Ethics approval and consent to participate

The Research Ethical Committee of Ghent University Hospital, Ghent, Belgium, approved this study (project number 2017/0129). Written informed consent is obtained from all participants prior to enrolment.

## Supplementary material

Supplementary material

## References

[1] Lee IM, Shiroma EJ, Lobelo F, Puska P, Blair SN, Katzmarzyk PT; Lancet Physical Activity Series Working Group. Effect of physical inactivity on major non-communicable diseases worldwide: an analysis of burden of disease and life expectancy. Lancet 2012 Jul;380(9838):219–29. 10.1016/S0140-6736(12)61031-9 22818936 PMC3645500

[2] Li J, Siegrist J. Physical activity and risk of cardiovascular disease--a meta-analysis of prospective cohort studies. Int J Environ Res Public Health 2012 Feb;9(2):391–407. 10.3390/ijerph9020391 22470299 PMC3315253

[3] Cillekens B, Huysmans MA, Holtermann A, van Mechelen W, Straker L, Krause N et al. Physical activity at work may not be health enhancing. A systematic review with meta-analysis on the association between occupational physical activity and cardiovascular disease mortality covering 23 studies with 655 892 participants. Scand J Work Environ Health 2022 Mar;48(2):86–98. 10.5271/sjweh.3993 34656067 PMC9045238

[4] Clays E, Lidegaard M, De Bacquer D, Van Herck K, De Backer G, Kittel F et al. The combined relationship of occupational and leisure-time physical activity with all-cause mortality among men, accounting for physical fitness. Am J Epidemiol 2014 Mar;179(5):559–66. 10.1093/aje/kwt294 24305575

[5] Coenen P, Huysmans MA, Holtermann A, Krause N, van Mechelen W, Straker LM et al. Do highly physically active workers die early? A systematic review with meta-analysis of data from 193 696 participants. Br J Sports Med 2018 Oct;52(20):1320–6. 10.1136/bjsports-2017-098540 29760168

[6] Holtermann A, Krause N, van der Beek AJ, Straker L. The physical activity paradox: six reasons why occupational physical activity (OPA) does not confer the cardiovascular health benefits that leisure time physical activity does. Br J Sports Med 2018 Feb;52(3):149–50. 10.1136/bjsports-2017-097965 28798040

[7] Amlani NM, Munir F. Does physical activity have an impact on sickness absence? A review. Sports Med 2014 Jul;44(7):887–907. 10.1007/s40279-014-0171-0 24668290

[8] Lahti J, Lahelma E, Rahkonen O. Changes in leisure-time physical activity and subsequent sickness absence: a prospective cohort study among middle-aged employees. Prev Med 2012 Dec;55(6):618–22. 10.1016/j.ypmed.2012.10.006 23064133

[9] van Amelsvoort LG, Spigt MG, Swaen GM, Kant I. Leisure time physical activity and sickness absenteeism; a prospective study. Occup Med (Lond) 2006 May;56(3):210–2. 10.1093/occmed/kqj026 16641504

[10] Gupta N, Dencker-Larsen S, Lund Rasmussen C, McGregor D, Rasmussen CD, Thorsen SV et al. The physical activity paradox revisited: a prospective study on compositional accelerometer data and long-term sickness absence. Int J Behav Nutr Phys Act 2020 Jul;17(1):93. 10.1186/s12966-020-00988-7 32690043 PMC7370435

[11] Kivimäki M, Head J, Ferrie JE, Singh-Manoux A, Westerlund H, Vahtera J et al. Sickness absence as a prognostic marker for common chronic conditions: analysis of mortality in the GAZEL study. Occup Environ Med 2008 Dec;65(12):820–6. 10.1136/oem.2007.038398 18611969 PMC2715845

[12] Andersen LL, Fallentin N, Thorsen SV, Holtermann A. Physical workload and risk of long-term sickness absence in the general working population and among blue-collar workers: prospective cohort study with register follow-up. Occup Environ Med 2016 Apr;73(4):246–53. 10.1136/oemed-2015-103314 26740688

[13] Gupta N, Bjerregaard SS, Yang L, Forsman M, Rasmussen CL, Rasmussen CD et al. Does occupational forward bending of the back increase long-term sickness absence risk? A 4-year prospective register-based study using device-measured compositional data analysis. Scand J Work Environ Health 2022 Nov;48(8):651–61. 10.5271/sjweh.4047 35894796 PMC10546616

[14] Lund T, Labriola M, Christensen KB, Bültmann U, Villadsen E. Physical work environment risk factors for long term sickness absence: prospective findings among a cohort of 5357 employees in Denmark. BMJ 2006 Feb;332(7539):449–52. 10.1136/bmj.38731.622975.3A 16446280 PMC1382535

[15] Clays E, Leynen F, De Bacquer D, Kornitzer M, Kittel F, Karasek R et al. High job strain and ambulatory blood pressure in middle-aged men and women from the Belgian job stress study. J Occup Environ Med 2007 Apr;49(4):360–7. 10.1097/JOM.0b013e31803b94e2 17426519

[16] Ralston K, Everington D, Feng Z, Dibben C. Economic Inactivity, Not in Employment, Education or Training (NEET) and Scarring: The Importance of NEET as a Marker of Long-Term Disadvantage. Work Employ Soc 2022;36(1):59–79. 10.1177/0950017020973882

[17] Edwards P, Greasley K. Health and well-being at work, working conditions. EurWORK. [Internet]. Ireland, Dublin: Eurofound; 2010, July 20 [cited 2023 Jul 6]. Available from: https://www.eurofound.europa.eu/publications/report/2010/absence-from-work

[18] Database of World Bank national accounts data, and OECD National Accounts data files, GDP (current US$), Belgium [Internet]. USA, Washington: The World Bank. [cited 2023 Jul 6]. Available from: https://data.worldbank.org/indicator/NY.GDP.MKTP.CD?locations=BE

[19] Database of Absenteeism from work due to illness, days per employee per year, Belgium. [Internet]. Copenhagen, Denmark: World Health Organization. [cited 2023 Jul 6] Available from: https://gateway.euro.who.int/en/indicators/hfa_411-2700-absenteeism-from-work-due-to-illness-days-per-employee-per-year/

[20] Holtermann A, Schnohr P, Nordestgaard BG, Marott JL. The physical activity paradox in cardiovascular disease and all-cause mortality: the contemporary Copenhagen General Population Study with 104 046 adults. Eur Heart J 2021 Apr;42(15):1499–511. 10.1093/eurheartj/ehab087 33831954 PMC8046503

[21] Maes I, Ketels M, Van Dyck D, Clays E. The occupational sitting and physical activity questionnaire (OSPAQ): a validation study with accelerometer-assessed measures. BMC Public Health 2020 Jul;20(1):1072. 10.1186/s12889-020-09180-9 32631292 PMC7339490

[22] Skotte J, Korshøj M, Kristiansen J, Hanisch C, Holtermann A. Detection of physical activity types using triaxial accelerometers. J Phys Act Health 2014 Jan;11(1):76–84. 10.1123/jpah.2011-0347 23249722

[23] Ketels M, De Bacquer D, Geens T, Janssens H, Korshøj M, Holtermann A et al. Assessing physiological response mechanisms and the role of psychosocial job resources in the physical activity health paradox: study protocol for the Flemish Employees’ Physical Activity (FEPA) study. BMC Public Health 2019 Jun;19(1):765. 10.1186/s12889-019-6950-7 31202266 PMC6570960

[24] Trost SG, McIver KL, Pate RR. Conducting accelerometer-based activity assessments in field-based research. Med Sci Sports Exerc 2005 Nov;37(11 Suppl):S531–43. 10.1249/01.mss.0000185657.86065.98 16294116

[25] Karasek R, Brisson C, Kawakami N, Houtman I, Bongers P, Amick B. The Job Content Questionnaire (JCQ): an instrument for internationally comparative assessments of psychosocial job characteristics. J Occup Health Psychol 1998 Oct;3(4):322–55. 10.1037/1076-8998.3.4.322 9805280

[26] Pelfrene E, Vlerick P, Mak RP, De Smet P, Kornitzer M, De Backer G. Scale Reliability and Validity of the Karasek Job Demand-Control-Support model in the Belstress Study. Work Stress 2001;15(4):297–313. 10.1080/02678370110086399

[27] The International Standard Classification of Occupations. 2008 (ISCO-08) (2012) Vol.1. Structure, group definitions and correspondence tables. International Labour Office, Geneva.

[28] López-Bueno R, Sundstrup E, Vinstrup J, Casajús JA, Andersen LL. High leisure-time physical activity reduces the risk of long-term sickness absence. Scand J Med Sci Sports 2020 May;30(5):939–46. 10.1111/sms.13629 31986220

[29] Wiese CW, Kuykendall L, Tay L. Get active? A meta-analysis of leisure-time physical activity and subjective well-being. J Posit Psychol 2018;13(1):57–66. 10.1080/17439760.2017.1374436

[30] Andersen LL, Clausen T, Persson R, Holtermann A. Dose-response relation between perceived physical exertion during healthcare work and risk of long-term sickness absence. Scand J Work Environ Health 2012 Nov;38(6):582–9. 10.5271/sjweh.3310 22714069

[31] Andersen LL, Pedersen J, Sundstrup E, Thorsen SV, Rugulies R. High physical work demands have worse consequences for older workers: prospective study of long-term sickness absence among 69 117 employees. Occup Environ Med 2021 Nov;78(11):829–34. 10.1136/oemed-2020-107281 33972376 PMC8526881

[32] Mirowsky J. 2017. Education, social status, and health. Routledge.

[33] Prince SA, Rasmussen CL, Biswas A, Holtermann A, Aulakh T, Merucci K et al. The effect of leisure time physical activity and sedentary behaviour on the health of workers with different occupational physical activity demands: a systematic review. Int J Behav Nutr Phys Act 2021 Jul;18(1):100. 10.1186/s12966-021-01166-z 34284795 PMC8290554

[34] Holtermann A, Marott JL, Gyntelberg F, Søgaard K, Suadicani P, Mortensen OS et al. Does the benefit on survival from leisure time physical activity depend on physical activity at work? A prospective cohort study. PLoS One 2013;8(1):e54548. 10.1371/journal.pone.0054548 23349926 PMC3547911

[35] Clays E, De Bacquer D, Janssens H, De Clercq B, Casini A, Braeckman L et al. The association between leisure time physical activity and coronary heart disease among men with different physical work demands: a prospective cohort study. Eur J Epidemiol 2013 Mar;28(3):241–7. 10.1007/s10654-013-9764-4 23329153

[36] Aadland E, Ylvisåker E. Reliability of Objectively Measured Sedentary Time and Physical Activity in Adults. PLoS One 2015 Jul;10(7):e0133296. 10.1371/journal.pone.0133296 26192184 PMC4508000

