# Supplementary material for: The impact of leisure-time physical activity and occupational physical activity on sickness absence. A prospective study among people with physically demanding jobs
Source: Scand J Work Environ Health. 2023 Oct 31;49(8):578–87. doi: 10.5271/sjweh.4120 (PMC10866622; doi:10.5271/sjweh.4120)
Supplement: Supplementary material [file SJWEH-49-578-S001.pdf]

The impact of leisure time physical activity and occupational physical activity on sickness absence. A prospective study among people with physically demanding jobs[1]

by Margo Ketels, MD,[2] Thomas Belligh, PhD, Dirk De Bacquer, PhD, Els Clays, PhD

1. Supplementary material
2. Correspondence to: Margo Ketels, Ghent University, Department of Public Health and Primary Care University Hospital Ghent, entrance 42 (4K3), Corneel Heymanslaan 10, 9000 Ghent, Belgium. [E-mail: Margo.Ketels@ugent.be]

Figure S1

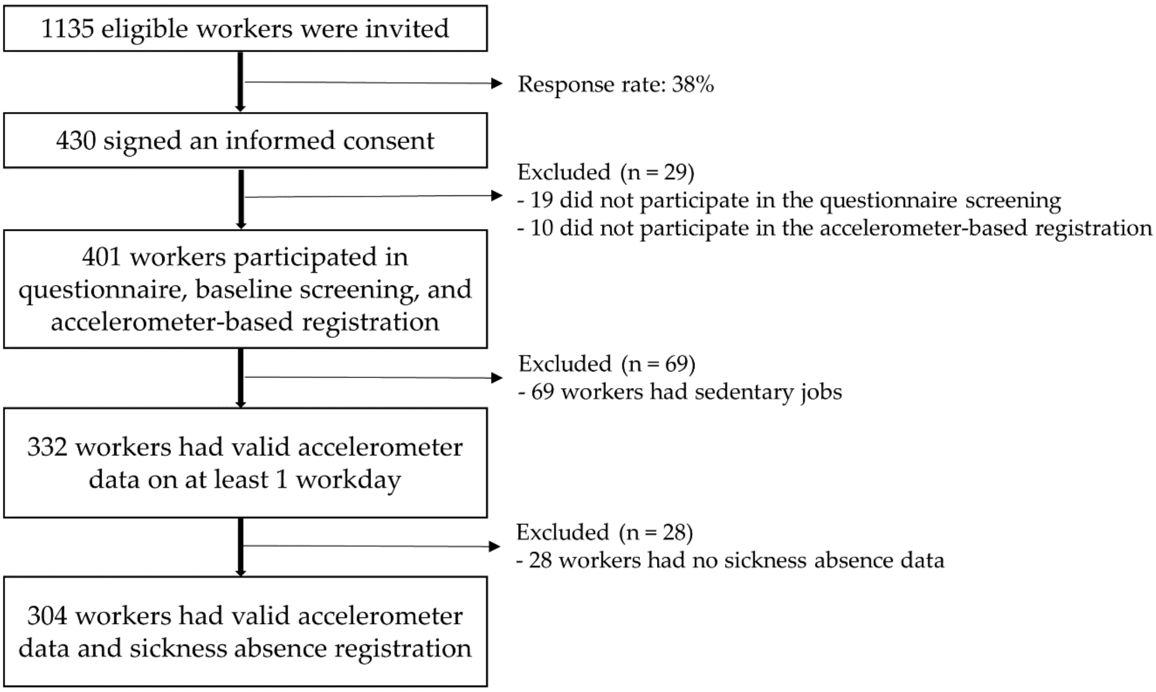

Supplementary Table S1. Negative binomial regression analyses: the association between OPA/LTPA and the number of sickness absence days. Fully adjusted model stratified according to education.

	Interaction	Low education		High education	
		Coeff	IRR (95% CI)	Coeff	IRR (95% CI)
MVPA work	p = 0.89	0.01	1.01 (0.98;1.04)	-0.01	0.99 (0.92;1.06)
Physical demands	p = 0.91	**0.50**	**1.65 (1.00;2.81)**	**0.59**	**1.80 (1.00;3.20)**
MVPA leisure	p = 0.21	-0.04	0.96 (0.90;1.03)	**-0.09**	**0.92 (0.85;0.99)**

Estimated model coefficients, incidence rate ratios (IRR) with 95% CI. Abbreviations: MVPA= moderate-to-vigorous physical activity; significant associations at p<0.05 are in bold. Adjusted for age, sex, BMI, smoking, and job strain.

Supplementary Table S2. Negative binomial regression analyses: the association between OPA/LTPA and the number of sickness absence periods. Fully adjusted model stratified according to education.

	Interaction	Low education		High education	
		Coeff	IRR (95% CI)	Coeff	IRR (95% CI)
MVPA work	p = 0.19	0.01	1.01 (0.99;1.03)	-0.01	0.99 (0.95;1.03)
Physical demands	p = 0.13	0.40	**1.49 (1.14;1.93**)	0.12	1.13 (0.81;1.58)
MVPA leisure	**p = 0.01**	0.01	1.01 (0.97;1.04)	**-0.06**	**0.93 (0.89;0.98)**

Estimated model coefficients, incidence rate ratios (IRR) with 95% CI. Abbreviations: MVPA= moderate-to-vigorous physical activity; significant associations at p<0.05 are in bold. Adjusted for age, sex, BMI, smoking, and job strain.

Supplementary Table S3. Negative binomial regression analyses: the association between OPA/LTPA and the number of sickness absence days. Fully adjusted model stratified according to job sector.

	Interaction	Service sector		Manufacturing sector	
		Coefficient	IRR (95% CI)	Coefficient	IRR (95% CI)
MVPA work	p = 0.38	0.02	1.02 (0.94;1.10)	-0.001	0.99 (0.97;1.03)
Physical demands	p = 0.75	**0.59**	**1.80 (1.00;3.16)**	**0.53**	**1.69 (1.04;2.81)**
MVPA leisure	p = 0.38	-0.07	0.93 (0.86;1.01)	-0.03	0.97 (0.90;1.05)

Estimated model coefficients, incidence rate ratios (IRR) with 95% CI. Abbreviations: MVPA= moderate-to-vigorous physical activity; significant associations at p<0.05 are in bold. Adjusted for age, sex, BMI, smoking, education and job strain

Supplementary Table S4. Negative binomial regression analyses: the association between OPA/LTPA and the number of sickness absence periods. Fully adjusted model stratified according to job sector.

	Interaction	Service sector		Manufacturing sector	
		Coefficient	IRR (95% CI)	Coefficient	IRR (95% CI)
MVPA work	p = 0.68	-0.003	1.00 (0.95;1.04)	0.002	1.00 (0.98;1.02)
Physical	p = 0.29	0.08	1.08 (0.77;1.52)	**0.47**	**1.60 (1.20;2.11)**
MVPA leisure	**p = 0.002**	**-0.06**	**0.93 (0.89;0.98)**	0.02	1.02 (0.98;1.06)

Estimated model coefficients, incidence rate ratios (IRR) with 95% CI. Abbreviations: MVPA= moderate-to-vigorous physical activity; significant associations at p<0.05 are in bold. Adjusted for age, sex, BMI, smoking, education and job strain

Supplementary Table S5. Descriptive characteristics of exposure and outcome variables for service and manufacturing sector separately.

	Service sector	Manufacturing sector
	Mean (SD) [min-max]	Mean (SD) [min-max]
Percentage MVPA at work	12.4 (5.01) [0-26.12]	17.1 (8.9) [2.4-39.6]
Percentage MVPA at leisure time	10.3 (5.28) [0-29.26]	9.1 (4.8) [1.3-27.4]
Physically demanding tasks	2.5 (0.68) [1-4]	2.4 (0.6) [1-4]
	Median (IQR)	Median (IQR)
Number of sick days during 1 year	0 (0-4)	4 (0.8-12.8)
Number of sick periods during 1	0 (0-1.5)	1 (0-2)

MVPA = moderate-to-vigorous physical activity
